# Supplementary material for: Acceptance and compliance with micronutrient powder (MNP) among children aged 6–23 months in northern Nigeria
Source: PLOS Glob Public Health. 2022 Oct 17;2(10):e0000961. doi: 10.1371/journal.pgph.0000961 (PMC10022258; doi:10.1371/journal.pgph.0000961)
Supplement: S1 Dataset — (ZIP) [file pgph.0000961.s008.zip › S8_Dataset/In-depth Interviews.zip!/In-depth Interviews/020715_K_BK_AB_2_CG.b.pdf]

I: Thank you very much for giving this opportunity to talk to you this morning. Can you describe to me the people in your immediate family living in your household

R: We are six

I: How many children do you have in this household that are between the ages of 6-23 months

R: Only this one (girl) with this baby

I: How old is she?

R: 19 months

I: What about this one

R: He is just about 5 months

I: We came here sometimes ago and we were told about food challenges, could you tell me about food challenges young children faced in this raining season

R: Although there are other food challenges we faced but for young children there is a problem of tea

I: How do you prepared tea

R: We make tea with Lipton, milk, bournvita and water

I: Which type of water hot or cold water?

R: Hot water that is boiling

I: Since you started using this MNP, could you tell me about your experience with the product

R: I have not started giving her

I: You mean you have not started using MNP what happen?

R: I wanted giving her father asks me to hold on first. So I have not started giving her is there lying down. And I was told you have to mix it with semi liquid (kunu), and she does not really like to take kunu. Apart from that, she stays in two places she is either here or there (other house) and this is ramadan period to really find food for her because of MNP is not easy. But we are hoping after the ramadan we will start giving her the MNP by then she will have been stable in staying in one house.

I: You mean after the fasting period you will start administering the MNP to her

R: Yes after the fasting, we will start administering the MNP to her

I: Thank you very much. Now that you have not started giving her the MNP where did you keep the product?

R: It is there in the room well kept.

I: There are some women that said it was hard to store the MNP because some children will come and steal it, some eaten by rodents. Tell me about your experience

R: I do not have such experiences except the very day when it was brought open as a sample for me to see nobody has temper with it

I: Although you said you have not stated using it, if this MNP is taken to the market would you purchase the MNP if it were cheaply on sale in your community?

R: If the people in the community are buying it, I will also follow and buy it

I: What do you think you will be willing to pay for each sachet?

R: I can afford to buy at two hundred naira (N200)

I: Thank you very much. Now please tell me some ways that you believe would be effective for promoting MNP to caregivers in this community.

R: You know I have not started using the product, so I will not know the effectiveness of it not to even encourage others to use it

I: In this very community who are the people when they talk people, they listen and obey their instruction

R: District head and our husbands

I: Where do you think would be good place to disseminate health information related to MNP

R: Like I told you I have not started using the MNP, so I will not know where its information should be disseminated

I: Who in your community do you trust the most for receiving messages about infant and young child feeding?

R: It is the father that usually takes her to clinic or hospital whenever she is sick, I don't usually go out to get any information

I: What forms of media do you prefer for receiving MNP messages?

R: You know this is a village setting unlike the city, which ever form you deem fit is welcome

I: You said you are yet to start using the product; do you have anything to say about the product?

R: I don’t have anything to say about the product, it is the father that said I should not give her yet.

I: Do you have any advice that you can give in respect to the product

R: I can only give advice had it been I have started using it and see its effectiveness then I can say something positive or negative about the product.

I: Okay, thank you very much.
